# Bioprocess Control: Current Progress and Future Perspectives

**DOI:** 10.3390/life11060557

**Published:** 2021-06-13

**Authors:** Anurag S. Rathore, Somesh Mishra, Saxena Nikita, Priyanka Priyanka

**Affiliations:** Department of Chemical Engineering, Indian Institute of Technology Delhi, New Delhi 110016, India; somesh25162@gmail.com (S.M.); saxena.nikita27@gmail.com (S.N.); priyanka.dalal24@gmail.com (P.P.)

**Keywords:** process monitoring, control systems, neural networks, fuzzy logic, automation, bioprocess control, open loop, closed loop

## Abstract

Typical bioprocess comprises of different unit operations wherein a near optimal environment is required for cells to grow, divide, and synthesize the desired product. However, bioprocess control caters to unique challenges that arise due to non-linearity, variability, and complexity of biotech processes. This article presents a review of modern control strategies employed in bioprocessing. Conventional control strategies (open loop, closed loop) along with modern control schemes such as fuzzy logic, model predictive control, adaptive control and neural network-based control are illustrated, and their effectiveness is highlighted. Furthermore, it is elucidated that bioprocess control is more than just automation, and includes aspects such as system architecture, software applications, hardware, and interfaces, all of which are optimized and compiled as per demand. This needs to be accomplished while keeping process requirement, production cost, market value of product, regulatory constraints, and data acquisition requirements in our purview. This article aims to offer an overview of the current best practices in bioprocess control, monitoring, and automation.

## 1. Introduction

Biopharmaceuticals or biologics have dominated the healthcare sector over the past decade [1]. This class of biotherapeutic products includes proteins, monoclonal antibodies (mAbs), and nucleic acids (DNA, RNA or antisense oligonucleotides) [2]. The success of these products is attributed to their effectiveness towards treating and managing a variety of otherwise hard to treat diseases including cardiovascular, neurological, cancer and other rare diseases. In 2017, the global market of biologics was USD 186.470 billion, and is expected to reach USD 526.008 billion by 2025 at a compound annual growth rate (CAGR) of 13.8% [3]. Within biotherapeutic products, mAbs are the most successful due to their success as therapeutics and for diagnostics [4]. The total global market of mAbs was over USD 122 billion in 2019, and is expected to surpass USD 200 billion in 2024 at CAGR of 6.9% [5].

Fundamentally, bioprocess development is performed while taking into account the nature of the host cell, microbial or mammalian. Often, the complex non-linear cellular growth and product kinetics are determined by the highly complex cellular metabolic network, ultimately impacting process yield and product quality [6,7,8,9]. The biologic manufacturing platform comprises of multiple upstream and downstream unit operations with the former including cell culture, cultivation, and harvesting unit operations, and the latter including multistep chromatography, filtration, and diafiltration [10]. 

Bioprocess productivity can be improved by efficient process control. Any control structure requires a sensor to detect deviation in a critical quality attribute (CQA) or a critical process parameter (CPP), monitoring recipe to decide when is it necessary to take action, and last but not the least, control logic for manipulating process variables to achieve the desired change and verification that the manipulation is effective and in right direction [11,12]. The past few decades have witnessed considerable contributions to process modelling, whether mechanistic, stochastic, or empirical [13,14,15]. Additionally, considerable advancements have been achieved in process control, with adaptive control, fuzzy logic, and neural networks to name a few. 

The selection of feedback control strategy depends on the complexity of system. If the system is just to be maintained at a static set point, then the use of computationally inexpensive proportional-integral (PI) control may be sufficient. More advanced control algorithms are needed for dynamic signal tracking or for control of complex systems. Irrespective of the computationally expensive nature of these algorithms, they have the potential to accurately capture the altercations of the desired metabolic pathways. These pathways contained complex regulation networks and thus show highly nonlinear behaviours. Recently, various digital approaches such as artificial intelligence (AI) for advanced monitoring and control and computational models have been implemented to study molecular or process-relevant behaviour [16,17,18].

Moreover, one of the guiding principles of quality by design (QbD) is to incorporate the use of modern control strategies and process analytical technology (PAT) tools so as to deliver consistent process performance and product quality [19,20]. QbD implementation involves identification of critical quality attributes (CQAs), comprising of physical, chemical, biological properties and other characteristics that need to be maintained in the desired limit, range or distribution [21]. Additionally, each CQA is influenced by critical process parameters (CPPs) whose fluctuations and effect over CQA must be supervised and control. PAT has been defined as “a system for designing, analysing, and controlling manufacturing through timely measurements (i.e., during processing) of critical quality and performance attributes of raw and in-process materials and processes, with the goal of ensuring final product quality” [22]. A key goal of PAT implementation is to design a process that can handle incoming variability to deliver consistent product quality [23]. 

In this review, we present a discussion of control strategies applied for control of bioprocesses. A variety of control schemes including feedback control, feedforward control, cascade control, and advanced control techniques have been addressed. It has been elucidated how an optimal control scheme aims to seek a compromise between bioprocess dynamics (well understood concepts) and bioprocess kinetics (less understood concepts). Challenges faced during implementing advanced control strategies are also highlighted. 

## 2. Sustainability in Biologic Manufacturing

Before discussing bioprocess control strategies, we would like to briefly explore the concept of sustainability in biologic manufacturing. The control strategies should be developed in a way that they complement sustainability. Over past decade, the perception of sustainability has evolved to describe such conditions that enable peaceful existence of industry with nature while meeting present and future generation socioeconomic demand [24,25]. In sustainable biologic manufacturing, the existing economic and environmental challenges need to be effectively managed. It requires an assessment framework comprising of qualitative indicators from life cycle assessment (LCA) and techno-economics analysis (TEA) to optimize biologic manufacturing at the preliminary stage itself [24,26]. It allows us to identify trade-offs across environmental and economic aspects over the entire process of biologic manufacturing. 

For decades, LCA is known as a standard approach in many commercial industries. However, its potential is yet to be fully realized in biologic manufacturing. Few studies have highlighted the importance of LCA and TEA for designing more cost-efficient, robust and environmentally friendly biologic manufacturing processes. In one study, Biosolve simulation tool has been implemented to conduct LCA analysis of the mAb manufacturing process. Here, the CIP and SIP steps are found to have greater environmental impact in the same study [26]. Another study assesses the process development and production cost across biopharmaceutical product cycle along with their contribution in overall research and development (R & D) cost [27]. The utilization of single use technology has been suggested to be economically favourable for short term and small-scale production (such as clinical production) [28]. For life cycle inventory, software such as Biosolve and Superpro Designer are utilized to model a new production line in combination with LCA software GaBi [29]. LCA in conjunction with TEA helps in accessing and optimizing sustainability performance in terms of environment and economics of biologic manufacturing. It allows us to identify hotspots (major environmental problems or costs) that may occur and how changes in life cycle inputs (resources used) and outputs (emissions into the environment) can reduce such hotspots. Further, LCA can take into account environmental impact of process as a whole which make it an ideal decision support tool to improve the decision-making quality in biologic manufacturing.

## 3. Strategies for Bioprocess Control

**Figure 1:**
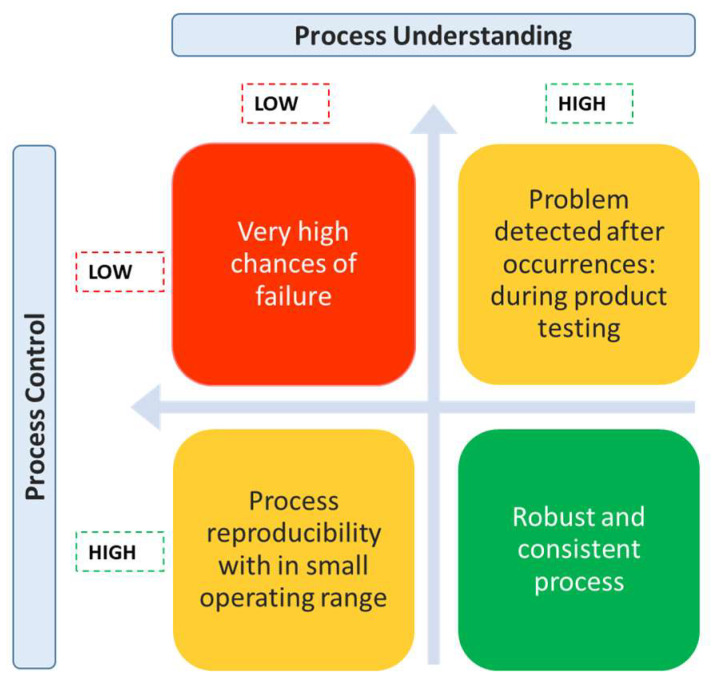
Importance of process understanding and controller for process development.

Bioprocess control comprises of a set of operations that supervise the process in an unpredictable environment with the objective to maintain the process within the desired design space. Typically, the control strategy is created during process development. Creation of a robust control strategy depends on how deep our understanding of the process is and if we have accurate process models. Inaccurate process models are known to result in instability of the controller [30]. Another major challenge is that of data availability as often limited data is available during process development [30,31]. There is a direct relation between the level of process understanding and the degree of robustness of the control system (Figure 1). Table 1 lists the different strategies implemented for bioprocess control along with their control structures. Depending on the requirements, one can decide on the optimal choice between selecting sophisticated instrumentation and complex control laws or simple control law with elaborate monitoring system. 

### 3.1. Open Loop Control 

This is the simplest and oldest control technique and has been employed for several carbon-limited fermentation processes at industrial scale [32]. Here, online measurements are not required and hence, the strategy is unable to dismiss any disturbance in the system. Researchers have utilized the open loop control strategy to reduce batch-to-batch variability [33]. The predefined operating condition, based on the initial operating parameters, acts as the input to the system. In numerous studies, predetermined exponential feed profiles (calculated based on initial conditions and growth kinetic parameters) are reported [33,34]. In recent studies, optimization of fed-batch operating conditions based on open loop strategy have been reported for recombinant lipase B, vaccine synthesis, ethanol, and poly (3-hydroxybutyrate-co-3-hydroxyvalerate) production [34,35,36,37,38].

While open-loop control strategies have been successfully used for bioprocess control, significant limitations exist including requirement of pre-computed knowledge of profiles, difficulty in mathematical formulation of non-linear systems, challenges with respect to real-time application, and requirement of complex databases of the process [32,33,34]. Another major limitation is the incompetency to take corrective measures if random disturbances occur during process operations [39].

### 3.2. Proportional Integral/Proportional Integral Derivative (PI/PID) Control 

Closed loop control systems, such as PI and PID, are designed to overcome the disadvantages of open loop control. In such a system, a feedback term is added for regulatory action to offer closed loop control. Hence, the control law becomes the sum of feedforward and feed backward terms. The most common closed loop algorithm is the PID controller, owing to its simplicity, robustness, and ease in tuning. Recently, researchers have developed a SISO nonlinear bioreactor model using real-time data of *E. coli* fermentation and employed internal model control based PID (IMC-PID) [40]. It is seen that the proposed IMC-PID method resulted in superior performance with minimum absolute error when compared to other methods [40]. The real-time investigation of closed loop depicted satisfactory tracking of the set point levels. Similar studies conducted using IMC-PID controller for temperature control in CSTR during ethanol production gave better results [41]. For another application involving improvement in biomass growth, combination of PI control with Generic Model Control (GMC) technique is implemented to fed batch cultivation of *E. coli* BL21(DE3). The proposed approach satisfactorily tracks the biomass profile with controller capable to maintain relevant growth conditions [42]. Feedback control loops such as flow feedback, pH feedback, or a combination of both have been applied for buffer preparation in inline conditioning and have shown significant potential [43,44]. For complex systems, PID control strategy has been modified as a non-linear gain in sequence with a linear PID. In case the error between the set point and the real-time process variable is zero, the system behaves as a linear system otherwise as non-linear system. Considerable performance improvement has been observed with the proposed approach in case of regulatory control and set point tracking in closed-loop system [45]. Additionally, feedback PID control loops are employed for control of microbial culture wherein the culture composition is the input variable and the actuator influence population dynamics. Actuators can be pH, dissolved oxygen, temperature or addition of inducer compounds that affects the organism’s fitness. It is seen that simple feedback loop has enabled high reproducibility of processes with quality product [46]. Until now, such strategies are rarely experimented, but have a promising future [47]. 

While PI/PID based control offers optimal performance for linear processes, it has limited ability to cope up with non-linear processes [39,48]. Since the underlying physicochemical processes for most complex biotech unit operations are non-linear, these dated controllers are not suitable to the dynamically changing conditions that are observed in most biological systems and, hence, modifications are required to make the controller effective for non-linear processes. 

### 3.3. Cascade Control 

Cascade control systems are comprehensively used in industry to improve the dynamic response of the systems. They minimize the effect of load disturbance that are received in the secondary or slave loop. Few studies have demonstrated their application for processes where the transfer functions of the primary and secondary loop are parallel [49,50]. Performance of the cascade control system is better than the conventional single loop control due to the presence of multiple sensors to measure conditions in a controlled process [51]. Cascade control strategy is required when the single loop control strategy fails to deliver satisfactory control output and secondary variable measurement is possible [52]. In a way, it can be said that cascade control strategy is one step superior to PI/PID control. 

Despite encouragement from the regulators, only a few academic researchers have tested the potential of cascade control in biopharma manufacturing. In one study, the bioreactor reproducibility both within and throughout culture stations (12 bioreactor vessels blocks) of AMBR 15 fermentation system (AMBR 15f) evaluated in fed-batch mode suggested cascade control strategy (air/stirrer/oxygen) as a better option for obtaining optimum cell growth [53]. Similarly, two-sided control loop for pH control is preferred in bioreactors [54]. Researchers have also attempted to control DO levels during aerobic fermentation using *Pseudomonas putida* mt-2 via cascade control [55]. Use of cascade-control to regulate DO levels during continuous fermentation for recombinant lipase B production resulted in 5.8 times higher productivity over fed-batch [34]. In another application, researchers demonstrated the effectiveness of cascade control for nullifying batch variation during biopharma production wherein the controller allowed short-time process disturbances (e.g., feed pump disturbances and antifoam spikes) to be resolved and yielded satisfactory batch reproducibility [56]. 

Advantages of cascade control that have been demonstrated include: (1) likely disturbance is distributed in the secondary loop where the corrective measures are taken without influence over primary loop; (2) the lag phase associated with the auxiliary process part is completely abated in the secondary loop and hence, improved the response of primary loop; (3) gain variation of the auxiliary part is subdued in the same loop; and (4) the secondary loop allows the primary controller to accurately regulate the mass or energy flow. Thus, applying the cascade control strategy for biotherapeutics production is likely to see increasing interest in the scientific community.

### 3.4. Model Predictive Control

Model predictive control (MPC) has been widely attempted for bioprocess optimization [57,58]. Its primary requirement is a predictive process model, through which the dynamic and static interactions among the input, output, and disturbance variables can be apprehended and the control estimate can be synchronized with the optimum set points calculations [59,60]. Successful implementation of MPC to track the variable trajectory [49,61] and to maximize process variables has been reported in biomanufacturing [62,63]. Owing to the complexities and variabilities in mammalian bioprocesses, non-linear MPC (NMPC) with dynamic models has been successfully applied but with larger computational time [64,65]. However, this large computational time (up to several minutes) is acceptable for such processes due to the lengthy process time (typically in weeks). In addition, NMPC employed to address parametric uncertainties using min-max optimization coupled with unscented Kalman filter (UKF) resulted in better performance and also dealt with sensor unavailability issue for fed batch processes efficiently [66]. Researchers have also used MPC for real-time control of quality attributes [67]. In multicolumn counter current solvent gradient purification (MCSGP) process, MPC strategy successfully tracked system periodicity and rejected disturbances. The results obtained were in agreement with the experimentally optimized profiles [68]. Apart from this, single input single output (SISO) MPC and multiple input multiple output (MIMO) MPC when used for online estimation and control of the fed batch reactor have demonstrated better results than proportional integral controller and feedback/feedforward controller. Steady state stabilisation in oscillating cell culture bioreactors can be achieved by implementing MPC designed based on cell population balance models [69]. 

As the essence of MPC is based on the process model, whether mechanistic or stochastic, its success is also heavily dependent on the model’s accuracy and its ability to handle the system disturbances [30,32,70]. Inaccurate process models are known to lead to faulty results [25,48]. In the case of data driven model, a large number of data points are required [48,57,71]. Further, the MPC approach is considered as computationally expensive in comparison to other control strategies, especially when optimization is required at each time step [69,72]. Thus, despite MPC being well established in chemical industries, its acceptance in biotech sector requires further development of robust process models.

### 3.5. Neural Network-Based Control 

Application of neural networks (NN) is already established to solve complex tasks in different engineering, medical, and other domains [73]. Since NN can effectively deal with non-linear systems and has adaptive learning, its application in bioprocess monitoring and control is becoming increasingly popular. Usually, data availability is the biggest limitation in designing neural network. However, few researchers have developed an alternative option to overcome the data limitation by generating artificial datasets (incorporating random noise to original datasets). Such approach is used in modelling and optimization of fed batch process for cyanobacterial C phycocyanin production [74]. The results obtained were in par with the neural network designed using large dataset. Recently, an adaptive neural network technique is devised for tracking control. The method possesses a self-learning capability which gives an added advantage in case of unavailability of prior data [75]. In a similar study, closed loop controller designed with a neural network estimator for a nonlinear process resulted in minimum tracking error when compared to conventional open loop methods even in case of perturbations and parametric uncertainties [39]. It is seen that the method gives better accuracy and is simple to design. Different variants of neural network are useful in different cases. Use of radial basis function neural network for fed batch bioreactor gave satisfactory performance in case of time varying parameters, uncertain non-linear disturbances and unmodeled dynamics [76]. Researchers have demonstrated the prediction of fungal biomass through Multiphase Artificial Neural Network (MANN) model during the lag, log, and stationary growth phase. The result indicates successful prediction of nonlinear features of fed-batch bioreactors via the MANN model [77]. Monitoring transient state performance using ANN has been shown to offer a better approach for controlling variables [78]. 

It is seen that trained neural networks can effectively evaluate and monitor process variables. ANN in combination with extended Kalman filter gives improved predictions in real time. This structure overlayed by model predictive control has great potential in non-linear process control [79]. In another study, MLP3 neural network has been introduced for controller output regulation and optimization. The strategy was experimentally validated on alpha 1-antitrypsin (A1AT, human recombinant protein) production in *Pichia pastoris* expression system under the control of alcohol oxidase (pAOX1) and resulted in a significant improvement in product yield [80]. Apart from its role in control, NN offers significant potential in developing model for different unit operations, thus enabling model-based control. 

Advantages of neural network over first principle or empirical modelling include: (1) NN’s are more capable of dealing with high non-linearity; (2) the probability of higher structural complexity makes them more descriptive as compared to empirical models; (3) their structure needs not to be predefined; (4) they offer greater flexibility in modelling; and (5) NN’s are less noise prone and can be applied to the systems of utmost uncertainty. 

### 3.6. Fuzzy Logic-Based Control

Fuzzy logic inquisitively integrates human experience and reasoning that is fundamental to design nonlinear controllers. In a recent publication, researchers have reviewed the evolution of fuzzy logic [81]. Fuzzy logic-based controllers have found practical applications in wider areas of energy, medicine, material, economics and pharmacology sciences. 

Application of fuzzy logic-based controllers for control and decision making in bioprocess industry is well established. Bioprocesses such as fermentation operations are complex and laden with various uncertainty factors. Therefore, the setup of optimum process parameters is necessary for achieving higher growth rates and productivity. Multiple researchers have demonstrated application of fuzzy logic Takagi Sugeno fuzzy controller for tracking control of bioprocess [82,83]. Here, the process was modelled using the TS fuzzy model followed by the use of fuzzy observer for designing controller. Two different control approaches were employed for output tracking, i.e., parallel distributed compensation control and fuzzy optimal control. It was seen that fuzzy control had lower root mean square error while dealing with the non-linearity of the system. Similar results were published for the purification of secondary metabolite optimization [84]. In addition, implementation of fuzzy feedforward control strategy for temperature control in fermentation [85] or product concentration control in enzymatic reactor [86] have proved to be efficient control logics for improving load rejections in non-linear process. An interesting application of fuzzy logic controller in combination with ANN is seen for fed batch cultures. In this study, ANN was used as a soft sensor to estimate the glucose concentration whereas fuzzy logic controller was implemented for controlling substrate addition. An improvement in estimation and control was observed with an error of 6% [87]. Several researchers have demonstrated applications of fuzzy control systems for control and optimization of bioreactor operations [88]. 

Fuzzy logic-based approaches have also been demonstrated to successfully explain the complexity of biological processes. The rules describing the expert process knowledge do not contain any complex mathematical equations and hence, are easy to understand. Thus, fuzzy systems can be considered as a special case of local modelling approach in which the input domain is distributed among different fuzzy regions described via multivariate membership functions [82]. However, the limitations associated with fuzzy logic systems are substantial and include inaccurate estimation of parameters due to fine tuning of rules, lack of adaptability for dynamic process state or minute process variables change that may have greater impact on overall process due to lack of learning. Thus, it is difficult to estimate continuous and independent optimization of operating variables via expert knowledge. These systems are more suitable for retaining distinct process space and quality aperture.

### 3.7. Model-Based Control 

Process models provide the foundation of advanced process monitoring, optimization, and control. Such models can be mathematical, statistical, or empirical. Effective monitoring and control of processes through the use of mathematical models has been demonstrated by multiple researchers [25,30,89]. One such framework named parametric optimization and control (PAROC), which has been implemented for biopharmaceutical purification process, was developed using model-based control techniques. The platform consisted of system identification and model analysis followed by designing multi parametric model predictive control and resulted in satisfactory control [90,91,92]. Same methodology when applied to small scale chromatography systems also tested successful for steady state operations and in rejecting uncertain perturbations [93]. In addition, when extended it was applicable to pressure swing adsorption and simulated moving bed systems [94]. Following a similar approach for capture chromatography in integrated continuous processes, mechanistic model was established with the adaptive model predictive controller for design and control of the capture steps [95].

For the bioreactor, a number of control strategies have been applied. For example, DO-stat control strategy [96,97,98,99,100,101] and Extremum seeking control strategy [102,103,104]. DO stat or pH stat control strategies are based on the concept of indirect feedback control. Here, a simple on–off controller manipulates indirect variables if direct variable deviates. In DO stat control, concentration of dissolved oxygen is maintained constant whereas in pH stat control, the pH is maintained constant. Extremum seeking control, developed based on process models, solves optimization problem as control problem. It enhances system capability to reject disturbances and thereby reduces sensitivity and downtime. A recent case study demonstrates model-based control of end-to-end continuous process for manufacturing monoclonal antibodies. The upstream process included bioreactor integrated with ATF and downstream process included protein A capture, viral inactivation, cation and anion exchange chromatography integrated together. The proposed control strategy was capable of automation of the process for optimal operation [105,106]. 

**Figure 2:**
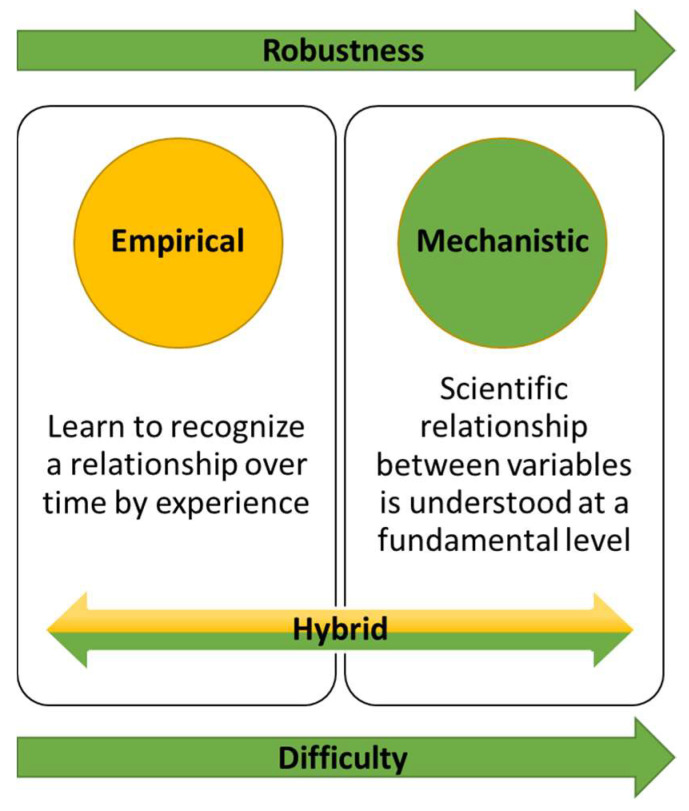
Example of hybrid model base bioprocess control.

However, realization of overall process supervision and control based on mathematical model remains an exception in biotech manufacturing. This is due to insufficient process understanding arising from process complexity. The mathematical models developed are therefore primarily used for elementary processes. In combination with archival data, real-time data, and observer intelligence have been used to develop hybrid models (Figure 2) [107,108,109,110]. Researchers have developed a holistic model from fundamental relations, transfer functions and the data from mAb process to simulate a high titre production bioreactor. Except for ammonia and glutamine, the application showed 80% agreement between predicted and experimental data [111]. Thus, holistic based control architecture can integrate static and dynamic feedback components along with logic based switching or discrete event elements. Despite the advancements in the fuzzy and NNs control systems design, a control system that can cope with uncertainty and nonlinearity remains overdue. However, most of the attention towards the development of hybrid controllers has been focused on set point control and tracking problems. 

## 4. Future Scope

### 4.1. Soft Sensor-Based Control

**Figure 3:**
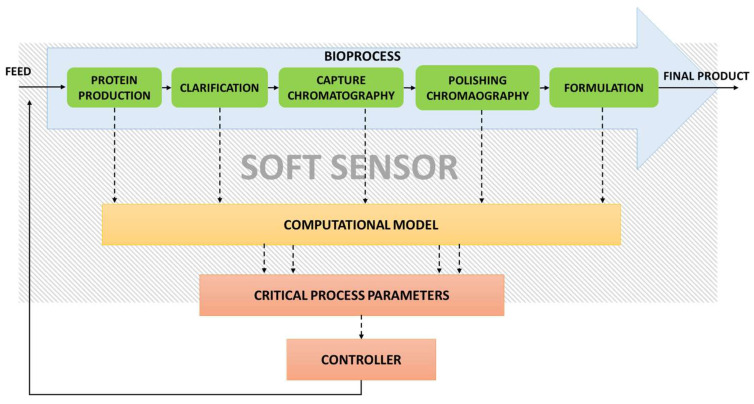
Soft sensor-based control set up for a generalised bioprocess.

Considering the complexity of bioprocesses, modelling to capture the overall characteristics and variability remains a challenge. In bioprocesses, due to limited availability of reliable on-line sensors, majority of process variables are evaluated off-line or at-line, thereby resulting in an increase in overall system cost and delivery of inefficient information. Moreover, operation and calibration for long duration in agitated, harsh environment for online sensors and manual operation with varying frequency of measurement for at line sensors are additional challenges in acquiring data. This has led to development of cybernetical-physical systems wherein the integrated physical systems are controlled by soft sensors and algorithms (Figure 3). Soft sensors have emerged as potential tools for evaluation and maintenance of CQAs in on-line mode, thus enabling QbD [112,113,114]. Different studies have shown the uses of soft sensors for determination of biomass, product, metabolites, amino acid concentration, and other CQAs, thus enabling process control (Table 2). Although advancements in the soft sensors development are vast, their real-time implementation requires further the development of non-invasive analytical techniques, with the ability to monitor in situ or in real time; sensor devices adaptable in various production systems and make it certain that the sensor configuration adheres to regulatory compliances and good manufacturing practices. Real-time, user-friendly interfaces are required so that the connection and contextualization of information from different sensors can be made possible via digitalization [115].

Advancements in miniaturization of sensors, development of smart sensors, and approaches for hardware-software integration (digital highway like fieldbus/profibus or wireless) has given an extra advantage to its adoption in manufacturing. Industry 4.0 requires computer algorithm based monitoring and cyber–physical system control. If the connections between soft sensors and process system engineering is investigated thoroughly and carefully, then it can be said that with soft sensors, Industry 4.0 can become a reality in bioprocessing with a promising future [131,132]. 

### 4.2. PAT Based Control Strategies

**Figure 4:**
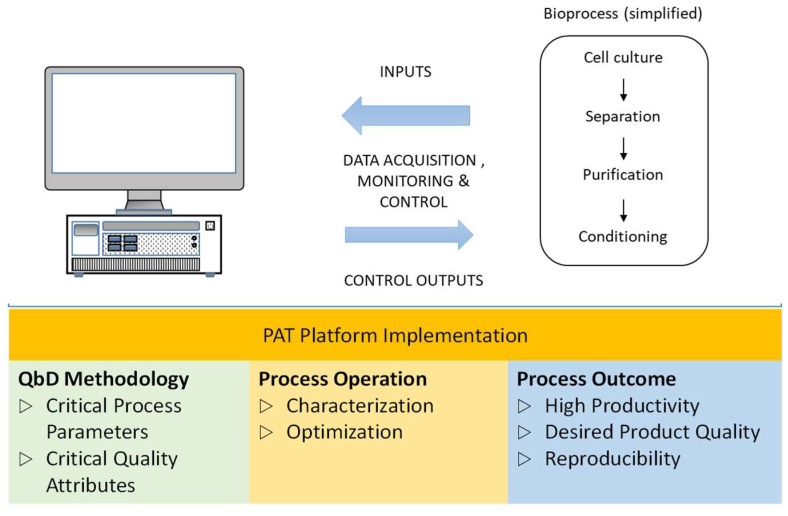
Bioprocess control implementation in the Quality by Design (QbD) paradigm.

The task of the controller is to manipulate the process variable in a way that the disturbance effect can be minimized and the process variable follows the specified trajectory. As discussed in the previous sections, traditional feedback PI controllers are widely implemented in the industries followed by the cascade control strategy. Advanced control strategies like multivariable control, model-based control, and adaptive control find limited industrial applications thus far. Despite major advancements that have been accomplished in the last two decades, more needs to be done to gain wider acceptance amongst manufacturers. The simultaneous use of ‘all’ available process information, its processing and convergence to meaningful action is a complex task and requires expertise. However, by integrating accessible process information at different levels of sophistication advance control schemes can be implemented. Based on the process understanding, advanced bioprocess control can be successfully applied (Figure 4) by integrating expert systems and artificial intelligence. Organized use of schematic information and logical process description gained by experience is key to success [107,109,110,111]. Understanding the relationship between the CQA (process outputs) and the CPP (measurable process operational variables) is essential for creating an effective control scheme [133], and performing real-time monitoring [134,135,136], as well as fault diagnosis [137].

**Figure 5:**
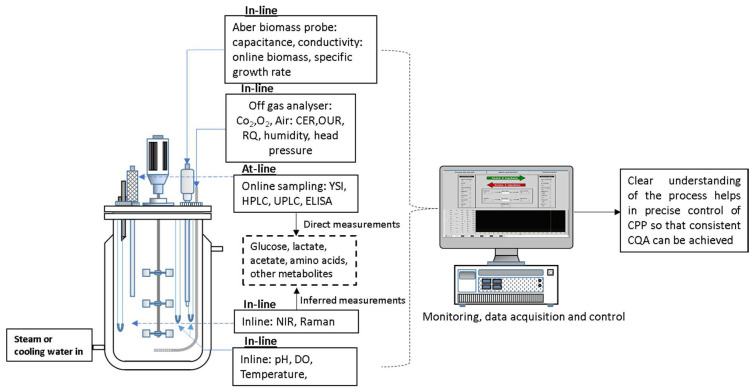
Example of advances in process monitoring for measurement of critical process parameter in bioreactor.

In recent times, PAT based methodologies have encouraged biopharmaceutical industries to change their modus operandi from quality by inspection to quality by design (QbD). However, the reluctance in industrial adoption is mainly due to the complex regulatory environment and issues faced in implementation of technology [138]. Additionally, for bioprocesses, high level of process understanding and control is required. Lately, spectroscopic PAT tools are gaining importance due to their ability to non-intrusively measure multiple process variables in real time. Additionally, spectroscopic tools can be used to screen cell culture media that helps in identifying correlations between the CPP and CQA’s [139]. The generated data can be processed via multivariate data analysis (MVDA). Figure 5 depicts an example of advanced monitoring of CPPs for a bioreactor, the data from which can be analysed and used for facilitating advanced process control. 

### 4.3. Automation in Biomanufacturing 

**Figure 6:**
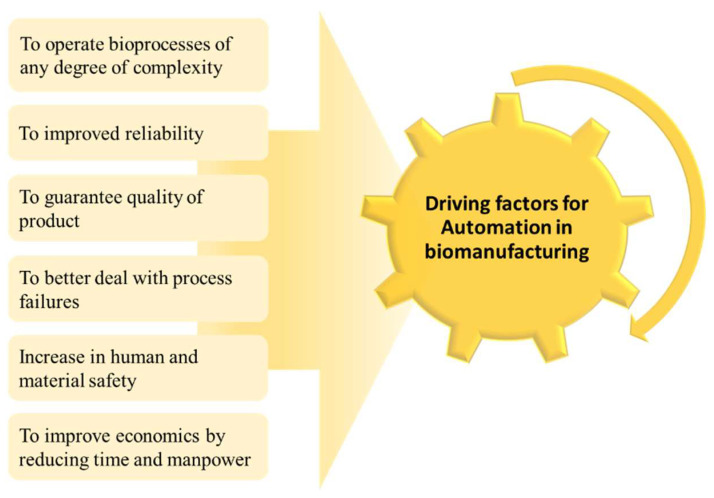
Benefits of automation in a biomanufacturing process.

Onset of automation in biomanufacturing is a key step towards robust process control (Figure 6). Digitalization is the new mantra with online sensors spewing continuous data on a multitude of variables [140]. Developments of inline and at-line sensors, wireless technology for connectivity of sensors to servers, smart sensors for acquiring data, and advancements in sensor calibration and compact technology are being increasingly used to address space limitations and logistic constraints. However, open platform communications or object linking and embedding approach can be implemented for integrating unit operations [141,142]. A key challenge that remains is that of incorporation of the automated and isolated workstation into the continuous workflow without affecting process efficiency. This includes consistency between different versions of the software, proprietary interfaces, and various data formats. Therefore, the primary need for successful integration is the use of standardized communication protocols and graphical user interfaces [136]. A critical criterion in the acceptance of middleware among users is scalability and flexibility. Integration of enterprise control system (ECS) into business systems (BS), manufacturing execution systems (MES), and shop-floor control systems (SFC) is the next stage of primary challenge to tackle when implementing a plant-wide information control system. Progress in integration-in-manufacturing through centralized/distributed hardware/software automation architectures is continuously growing via intelligence-in-manufacturing epitome addressed by industry centric R & D activities. As an example, supervisory control and data acquisition (SCADA) platform is implemented for an end-to-end integrated process wherein the system integrates and analyses different unit operations and collects and stores data to enable monitoring and control [143]. Additionally, the concept of digital twin for enabling control is shown for small scale end-to-end monoclonal antibody production platform [105]. However, full potential of automation in end-to-end bio-manufacturing process at industrial scale is yet to be realized. 

Another significant challenge revolves around data analysis. Bioprocess analytics is recognized as a crucial technical barrier to the acceptance of bio-manufacturing automation. Research labs are producing an increasing amount of data, both in quantity and complexity, which requires further analysis [144,145]. Further, the regulatory agencies are focused on issues around data integrity [144]. Therefore, operable and industry standard software systems are needed for data management. In addition, bio-manufacturing systems must be designed such that they can handle the high complexity of the data and have sufficient agility and flexibility. 

## 5. Conclusions

In the last two decades, tremendous enhancement in process productivity has been realized. Advancements in process control and monitoring have helped in reducing development costs and improving affordability. Considering the associated complexity and inconsistency, manufacturing of products like mAbs poses a major challenge to conventional production practices. Inspired by the recent regulatory guidelines within the QbD framework, stochastic and mechanistic model-based controllers are now emerging as popular choices for bioprocess control. Contrary to the traditional experimental approach, it is observed that the utilization of simulations and advanced statistics results in a low cost and in a shorter time. This has led to the development of process control strategies, from cascade to adaptive to hybrid, along with introduction of neural network (NN)-based controllers. Recent efforts have been made to conceptualize holistic process model-based controllers that offer a digital replica of the end-to-end bioprocess. It involves integration of individual unit operation along with monitoring and control, providing deeper insight on the impact of complex correlations between CPPs and CQAs for coupled unit operations. At present, model-based controllers are capable of contributing towards root cause analysis, molecular interactions, and refinement of unit operation models. However, they typically have high computational burden. In recent times, neural network-based control strategies have made significant progress, but their use in process modelling requires extensive datasets. Hence, an integrated approach combining statistical models with detailed theoretical models is required to avoid comprehensive experimentation and obtain deeper understanding. Thus, control strategies need to be designed aiming to achieve high levels of precision, accuracy, and robustness. 

In addition, automation in manufacturing would provide substantial opportunities to overcome many of the challenges faced in commercial success. However, major technical and business strategy challenges are encountered while creating scalable and automated bio-manufacturing solutions. Thus, biotherapeutic developers must think from the perspective of large-scale production and should incorporate automation concepts from the earliest stage of process development. Inadequacy of standardization across software, hardware and design specification complicates the attempts of automation. To overcome these barriers, manufacturers must continue to increase their process understanding and utilize this understanding to develop a streamlined and efficient bio-manufacturing process featuring validated in-process testing and control. Last but not the least, stakeholders and technical solution providers should attempt to meet the innovation gap in biomanufacturing and liaise jointly with biotherapeutic developers to design and develop automated solutions.

## Figures and Tables

**Table 1:** Description of different control strategies and their control structures.

Control Strategy	Description	Control Structure
Open loop control	Pre-computed and sequential control actions are stored in a controller and executed on demand Control action cannot be adjusted based on system response/disturbance Open loop control strategy can only give instruction to the equipment No means of data acquisition	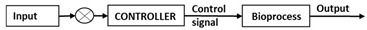
Closed loop control/ feedback control with PI/PID controller	System response/outputs are continuously compared with the set point In case of deviation, controller output is adjusted to minimize the error and forcing response towards the set point Employed for more challenging operations such as automation for continuous production of biotherapeutics	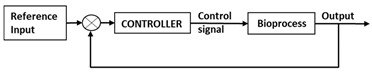
Cascade control	Input to the primary loop process transfer function is obtained from the output of secondary loop process transfer function The presence of “secondary” measured process input variable checks the emergence of primary disturbance. This improves controller performance by lowering both the maximum deviation and the integral error for disturbance responses	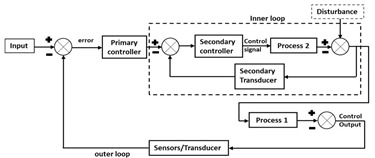
Fuzzy logic-based control	Flexible reasoning method that imitates human decision making based on IF- ELSE protocol It comprises of 4 modules: Fuzzification, Knowledge based, Inference system, and Defuzzification Can be applied in case of imprecise domain knowledge with distorted and noisy data Limitation includes dependency on human knowledge and experience, low accuracy, no systematic approach to problem solving, and requirement of regular updates of fuzzy rules for the controller to remain effective	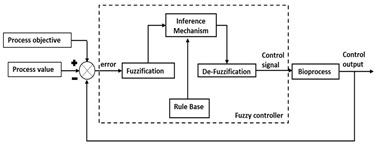
Artificial neural network-based control	Comprises of input layer, hidden layer, and output layer Hidden layer has weights that transform input into a quantity that can be used by output layer. The technique adjusts hidden layer to match the desired output. NN are designed for pattern recognition, classification, clustering, and prediction. Limitations include the extensive amount of data required and computational time	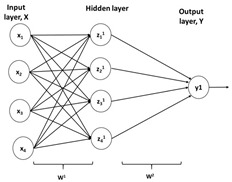
Model predictive control	Multivariate control algorithm that uses real-time process model for making predictions by optimizing cost function at each step to reach the reference value Improves steady state response, predicts upcoming disturbances, and guarantees prediction stability Requires accurate real-time model and the computational cost is high	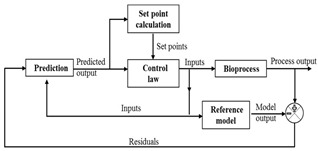
Model based control	Model based control is implemented in 4 steps: plant modelling, analysing and developing controller, simulating plant, and controller Simple and intuitive design with better performance than PID	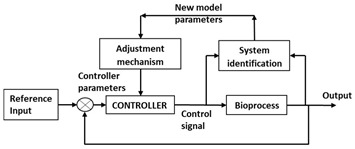

**Table 2:** Cases studies on soft sensor development for various applications.

Technique	Application	Reference
Artificial neural network	Monitor fermentation process—measurement of glycerol, 1,2 propanediol and biomass	[116]
Multiple linear regression in combination with mechanistic model	Prediction of biomass concentration	[117]
Empirical models	Biomass concentration	[118]
Empirical modelling of oxygen uptake rate	Predicted viable biomass	[119]
Multivariate adaptive regression spline algorithm in combination with 2D fluorescence spectra and process data	Biomass concentration	[120]
Artificial neural network	Glucose estimation in fed batch culture	[78]
Deep neural network	Parameter estimation in penicillin and streptokinase fermentation process	[121]
Partial least square regression with turbidity and Raman measurements in combination with Monod kinetics model	Predict substrate concentration	[122]
Error propagation method	Estimate error in predicted biomass fermentation rate and substrate consumption rate	[123]
Partial least square regression in combination with fluorescence fingerprinting	Monitor biotransformation production of 2 phenyl ethanol by yeast	[124]
Partial least squares regression model in combination with Raman spectroscopy	In line monitoring of the nutrient consumption and production of markers associated with cell metabolism	[125]
Partial least squares regression model in combination with Raman spectroscopy	In line monitoring on amino acid	[126]
Partial least squares regression with the help of mid-UV absorption spectra	Monitor chromatography steps	[127]
Partial least squares regression with Fourier transform mid infrared spectroscopy	Monitor HCP and aggregates at line	[128]
Deep neural network	Lactose and ethanol concentration measurement	[129]
Linear regression on data obtained from biocalorimetry in combination with bioreactor off gas analysis	Biomass concentration measurement	[130]

## Data Availability

Not applicable.
